# Longitudinal Impact of APRNs on Nursing Home Quality Measures in the Missouri Quality Initiative

**DOI:** 10.1007/s12603-021-1684-5

**Published:** 2021-10-01

**Authors:** Marilyn Rantz, G. F. Petroski, L. L. Popejoy, A. A. Vogelsmeier, K. E. Canada, C. Galambos, G. L. Alexander, C. Crecelius

**Affiliations:** 1 University of Missouri Sinclair School of Nursing, Columbia, Missouri USA; 2 Biostatistics Unit, School of Medicine, University of Missouri, Columbia, USA; 3 University of Missouri, Columbia, USA; 4 University of Wisconsin-Milwaukee, Milwaukee, WI USA; 5 Columbia University School of Nursing, Columbia, USA; 6 Post-Acute, BJC Medical Group, Washington University School of Medicine, St. Louis, USA; 7 University of Missouri Sinclair School of Nursing, Columbia, USA

**Keywords:** Nursing homes, quality of care, QMs, APRNs, nursing home payment

## Abstract

**Objectives:**

To measure the impact of advanced practice nurses (APRNs) on quality measures (QM) scores of nursing homes (NHs) in the CMS funded Missouri Quality Initiative (MOQI) that was designed to reduce avoidable hospitalizations of NH residents, improve quality of care, and reduce overall healthcare spending.

**Design:**

A four group comparative analysis of longitudinal data from September 2013 thru December 2019.

**Setting:**

NHs in the interventions of both Phases 1 (2012–2016) and 2 (2016–2020) of MOQI (n=16) in the St. Louis area; matched comparations in the same counties as MOQI NHs (n=27); selected Phase 2 payment intervention NHs in Missouri (n=24); NHs in the remainder of the state (n=406).

**Participants:**

NHs in Missouri Intervention: Phase 1 of The Missouri Quality Initiative (MOQI), a Centers for Medicare and Medicaid (CMS) Innovations Center funded research initiative, was a multifaceted intervention in NHs in the Midwest, which embedded full-time APRNs in participating NHs to reduce hospitalizations and improve care of NH residents. Phase 2 extended the MOQI intervention in the original intervention NHs and added a CMS designed Payment Intervention; Phase 2 added a second group of NHs to receive the Payment. Intervention Only.

**Measurements:**

Eight QMs selected by CMS for the Initiative were falls, pressure ulcers, urinary tract infections, indwelling catheters, restraint use, activities of daily living, weight loss, and antipsychotic medication use. For each of the monthly QMs (2013 thru 2019) an unobserved components model (UCM) was fitted for comparison of groups.

**Results:**

The analysis of QMs reveals that that the MOQI Intervention + Payment group (group with the embedded APRNs) outperformed all comparison groups: matched comparison with neither intervention, Payment Intervention only, and remainder of the state.

**Conclusion:**

These results confirm the QM analyses of Phase 1, that MOQI NHs with full-time APRNs are effective to improve quality of care.

## Introduction

In 1965, the first Nurse Practitioner program opened at the University of Colorado (1). APRNs began practicing in nursing homes (NHs) in the 1970s (2) and studies about their positive impact on NH care began to emerge in the 1980s (3, 4, 5). Four systematic reviews examining forty years of studies about the effectiveness of APRN care for NH residents demonstrate consistent results: improved resident health and functional status; reductions in hospitalizations, emergency room admissions and costs of care; improved resident quality of life; and improved resident and family satisfaction with care (5, 6, 7, 8). There is a strong foundation for encouraging APRN practice in NHs to systematically improve resident quality of care.

The Missouri Quality Initiative (MOQI), a Centers for Medicare and Medicaid (CMS) Innovations Center funded research initiative, was a multifaceted intervention in NHs in the Midwest, which embedded full-time APRNs in participating NHs (9). The MOQI was one of seven sites the national demonstration, Initiative to Reduce Avoidable Hospitalizations among Nursing Facility Residents (10).

The Initiative began in Phase 1 (2012–2016) with the goals to reduce unnecessary hospital and emergency department transfers; improve resident health outcomes; improve the process of transitioning between inpatient hospitals and NHs; and reduce overall healthcare spending without restricting access to care or choice of providers. External evaluators of the Initiative, after analyzing three years of quantitative data (Medicare claims and other NH assessments) compared with six other state sites, reported that MOQI interventions were associated with a consistent and significant reduction in the key outcomes (11). The results of quantitative analysis of key outcomes of the MOQI intervention of Phase 1 were reported (11–13). Also, an analysis of Quality Measures (QMs) during Phase 114 revealed a composite of eight QM scores of the MOQI APRN intervention group that were significantly better (P = .025) than a matched comparison group (14). The eight QMs selected by CMS for the Initiative as key measures of quality of care in long-stay residents were falls, pressure ulcers, urinary tract infections, indwelling catheters, restraint use, activities of daily living, weight loss, and antipsychotic medication use. QMs were developed in the 1990s as measures of quality of care (15). Since 2002, these measures are publicly reported to assist consumers in locating NHs with better quality of care (16).

The MOQI Initiative continued in Phase 2 (2016–2020) to test the effect of a Payment Intervention on hospitalizations and costs for NHs who were selected to receive payment for additional care of acutely ill residents. During Phase 2, the APRNs continued to work in their assigned NHs (n=16) as they did in Phase 1, with the addition of the Payment Intervention. As in Phase 1, the MOQI APRNs used their NH’s QMs to guide educational programs and quality improvement efforts. A new group of NHs without MOQI APRNs (n=24) also implemented the same Payment Intervention.

The purpose of this article is to extend the Phase 1 analysis (14) of the impact of APRNs on the QM scores of the 16 MOQI NHs over both Phases 1 and 2 (September 2013 through December 2019), six years of full implementation of the MOQI intervention. (Note: 2020 was a partial year of Phase 2 and not included in this analysis due to the impact of COVID-19 pandemic). During both Phases, APRNs focused on quality improvement strategies with potential to influence healthcare outcomes. Additional analyses evaluated the impact on care quality of full time APRNs on QM outcomes of the MOQI NHs and compared scores to other groups in the state, including the Payment Only Intervention group.

## Sample

Monthly QM reports for Missouri NHs LTC were provided by the state’s Quality Improvement Organization under appropriate data use agreement (DUA). The data span the time frame from September 2013 thru December 2019. NHs with incomplete data were excluded from the analysis.

There are four groups of NHs for this analysis. The groups labeled “CMS-B” and “B-Controls” are the same as in the Phase 1 report. CMS-B NHs are the original MOQI intervention group (n=16) with APRNs. B-Controls (n=27) is the original Phase 1 control group comprised of NHs in the same counties as the 16 intervention NHs, and had similar baseline QM scores, size, and ownership; further selection details of this group are published in the Phase 1 analysis (14). The “CMS-A” group (n=24) are NHs participating in the Phase 2, Payment Only intervention. CMS-A NHs were recruited by the MOQI team from an approved list of potential NHs provided by CMS. The final comparison group (n=406) is composed of Missouri homes not in the other three groups. In the following charts this group is labeled “ROTS”, an abbreviation for “Remainder of The State.” NHs with incomplete data on the long-stay QMs were excluded from the analysis. The exclusions (n=88) for missing and incomplete data were limited to the ROTS group. There were no missing data for the three groups of primary interest. The time for the weight loss QM was truncated at October of 2018 due to a change in definition for the QM (17).

## Methods

The outcome data consists of the Centers for Medicare and Medicaid Services Quality Measures (QMs) for long-stay NH residents. Statewide QMs were available to the research team under appropriate Data Use Agreement and other publicly available NH descriptive data of NH size, ownership, and location. The measures are reported monthly and span September 2013–December 2019. For each QM, four time series were created by averaging the monthly QM values over NHs within each study group. Preliminary analyses revealed a pronounced seasonal aspect for some QMs, and that all QMs exhibit very strong serial correlation between months. This last aspect of the QM data is inevitable given that a QM value for a given month is a rolling average of the current month and the five previous months. The length of the data series (76 months), the presence of seasonal effects, and autocorrelation are the features of the data that prompted the use of time series techniques.

For each of the QMs an unobserved components model (UCM) (18) was fitted. The full UCM decomposes a time series into additive components for trend, seasonal, cyclical (recurrence patterns without a fixed period), regression effects, and an “irregular component” (random error). Seasonal effects were modeled as a trigonometric series with an annual period. An additional feature of the UCM is that the irregular component can incorporate considerable structure such as autoregressive (AR) and moving average (MA) terms of different orders, and seasonal AR and MA effects as well. For each QM an initial UCM was fit to a time series for the entire State and the optimal irregular component, and for outcomes with a season component, the number of harmonics to retain, were selected via the Akaike Information Criterion (19). The selected model was then refit to the series for each group. The logic in fitting an initial model to data from the entire state is that averaging over all homes will produce monthly estimates with lower variance than estimates within any single group of NHs, thus making it easier to identify seasonality and harmonics, and correlation structures in the irregular component. Furthermore, these two features of the data are unlikely to be impacted by the interventions. Non-seasonal trend effects were allocated to the regression component and none of the models include a cyclical component. Trend for each QM was estimated as a piece-wise linear model with the possibility of a smooth transition in slope starting with the initiation of Phase 2 (January 2017) of the study. A linear trend is also fit for NHs comprising the remainder of Missouri. Phase 2 marks the start of the payment intervention and is not a relevant transition point for homes in the ROTS group, thus, only a single slope estimate was used for those NHs. Histograms and normal plots were used to evaluate the normality of residuals.

The analysis describes QM trajectories for each of the four groups. The CMS-A group did not exist at the beginning of the study; however, the trajectory of those NHs was constructed for Phase 1 to facilitate a pre-post type analysis for the evaluation of the payment intervention. Four of the NHs identified by CMS for the payment intervention were also in the B-control group. These four NHs were removed from the control and included in the CMS-A group for Phase 2.

## Results

Table 1 summarizes descriptive statistics for each QM with the mean, standard deviation, median, minimum and maximum QM scores for each group; it also includes certified number of beds in each group and percentage of for profit NHs for descriptive comparison. CMS-B had the largest average bed-size (166.6 vs 149, 140 and 100); it also had the largest percentage of for-profit NHs (87.5% vs 85.5%, 79% and 82%).

**Table 1:** Baseline QM Scores and NH Characteristics

**Group**	**Variable**	**Mean**	**SD**	**Median**	**Minimum**	**Maximum**
CMS-B	Falls	3.4	2.3	3.5	0.0	8.6
N = 16	Pressure Ulcer	6.4	3.8	6.1	1.3	14.1
	UTI	7.1	5.7	6.3	0.7	19.3
	Urinary Catheters	2.2	1.7	2.3	0.0	5.8
	Restraints	1.2	3.0	0.0	0.0	11.8
	ADL	12.5	7.6	13.1	0.0	24.6
	Weight Loss	9.0	4.2	8.8	2.2	18.8
	Antipsychotics	17.6	7.1	16.4	8.9	33.6
	QM Composite	7.3	1.8	7.6	4.3	11.1
	Certified Beds	166.6	61.5	151.0	89.0	321.0
	% for profit	87.5				
B-Controls	Falls	3.7	2.3	3.4	0.0	10.0
N = 27	Pressure Ulcer	5.8	2.9	6.7	1.1	12.8
	UTI	6.5	5.7	4.1	0.0	17.6
	Urinary Catheters	2.5	2.1	2.1	0.0	8.3
	Restraints	1.0	1.4	0.5	0.0	4.5
	ADL	12.4	7.4	11.0	1.9	28.8
	Weight Loss	10.0	4.4	10.8	1.3	17.8
	Antipsychotics	18.1	6.8	16.7	7.8	37.2
	QM Composite	7.2	1.6	7.3	4.5	10.2
	Certified Beds	148.9	56.2	130.0	90.0	310.0
	% for profit	85.2				
CMS-A	Falls	3.9	2.3	3.5	0.0	10.0
N = 24	Pressure Ulcer	6.2	4.9	5.3	0.0	20.0
	UTI	5.6	4.9	4.1	0.0	15.7
	Urinary Catheters	3.2	3.0	2.9	0.0	12.0
	Restraints	0.6	1.6	0.0	0.0	6.8
	ADL	13.1	8.2	10.6	1.1	26.0
	Weight Loss	9.3	5.3	10.2	1.1	19.0
	Antipsychotics	22.7	14.0	21.0	5.0	75.8
	QM Composite	7.7	2.1	7.6	3.2	13.2
	Certified Beds	139.9	49.5	122.0	90.0	314.0
	% for profit	79.2				
ROTS	Falls	4.2	3.3	3.6	0.0	17.6
N = 406	Pressure Ulcer	6.6	6.0	5.6	0.0	32.0
	UTI	6.9	6.0	5.8	0.0	37.5
	Urinary Catheters	3.4	3.1	2.8	0.0	18.5
	Restraints	1.2	3.1	0.0	0.0	42.1
	ADL	14.4	9.2	13.1	0.0	44.4
	Weight Loss	7.4	5.8	6.5	0.0	38.9
	Antipsychotics	24.2	13.1	21.7	0.0	87.5
	QM Composite	8.1	2.4	8.1	1.5	18.0
	Certified Beds	100.3	43.1	96.0	20.0	353.0
	% for profit	82.1				

UTI=Urinary Tract Infection; ADL=Activities of Daily Living; QM = Quality Measures; CMS-B=MOQI Intervention with APRNs (Phases 1 and 2) + Payment Intervention (Phase 2); B-Controls=Matched Comparison to CMS-B, No Intervention; CMS-A=Phase 2 Payment Intervention Only; ROTS=Remainder of the state (Missouri)

### Plots of Individual Quality Measures

**Figure 1:**
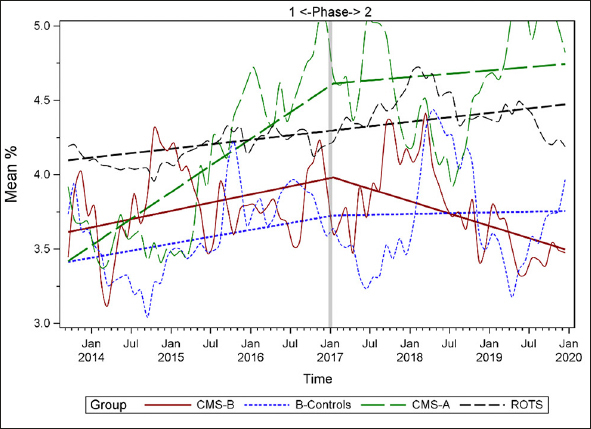
Residents experiencing one or more falls with injury

**Figure 2:**
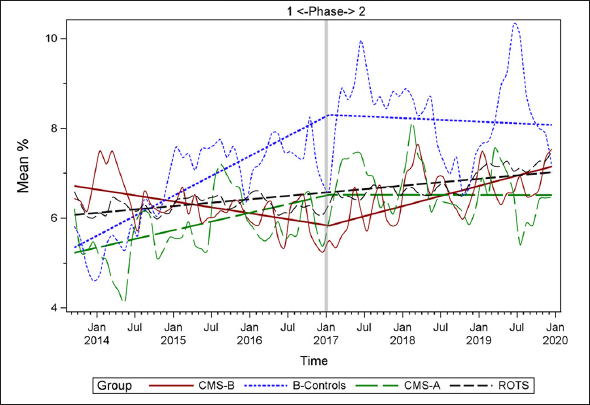
Percent of high-risk residents with pressure ulcers

**Figure 3:**
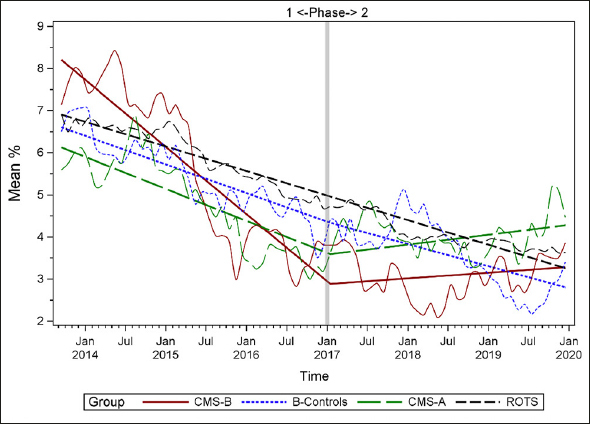
Residents with a urinary tract infection

**Figure 4:**
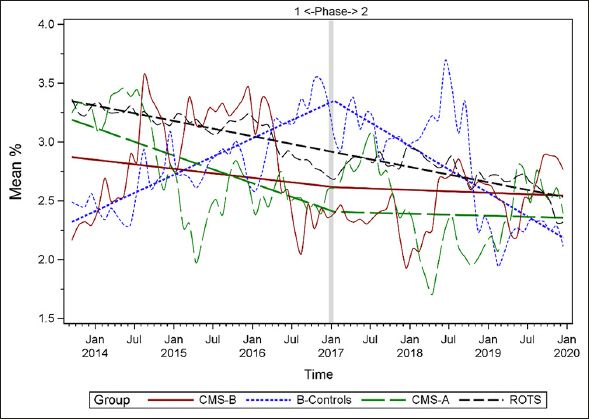
Residents who have/had a catheter left in their bladder

**Figure 5:**
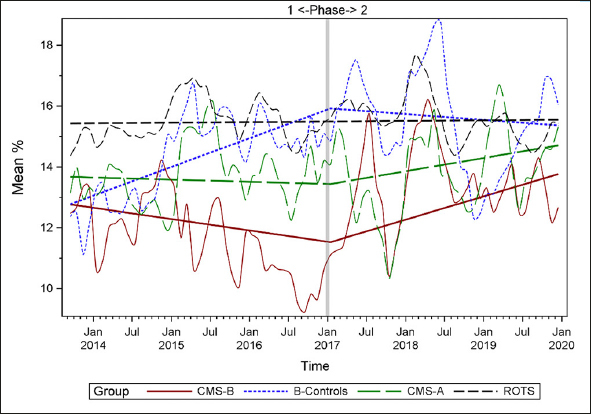
Percent of residents needing increased help with ADLs

**Figure 6:**
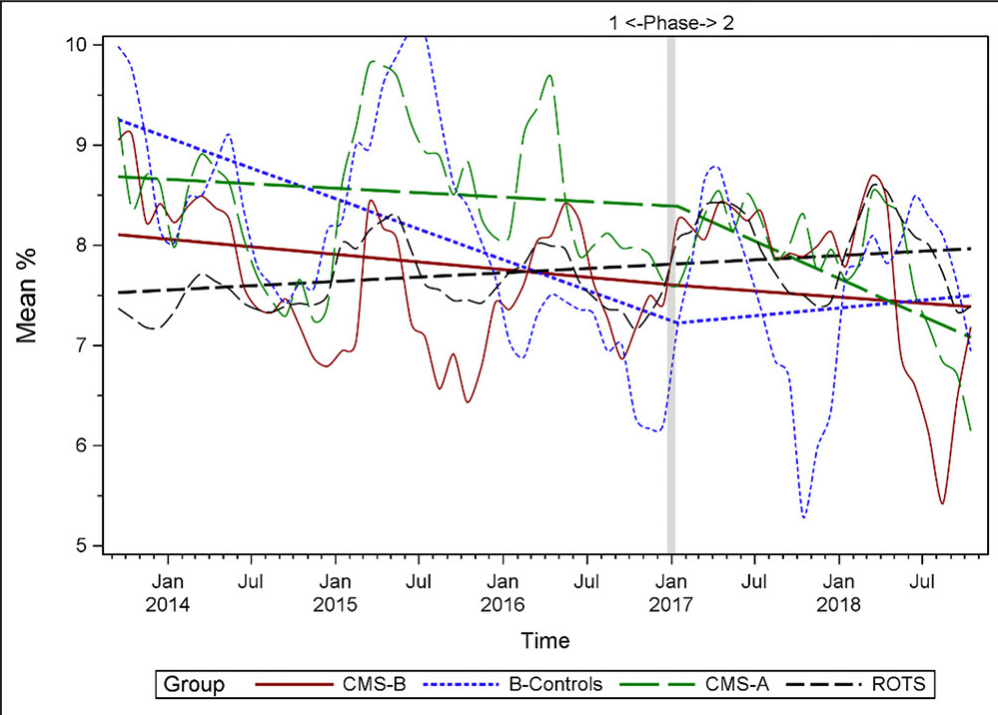
Residents who loose too much weight

**Figure 7:**
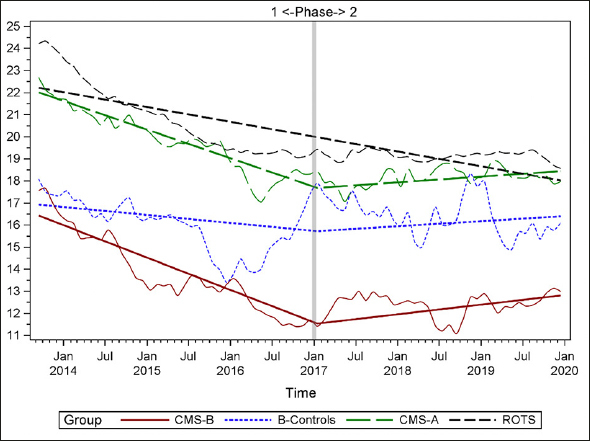
Long-stay residents receiving an antipsychotic medication

Figures 1–7 display seven of the eight QM scores over the course of the study. Phase 1 (2013–2016) tested the effectiveness of the MOQI intervention with APRNs and Phase 2 tested the effectiveness of the Payment Intervention (2017–2019). During Phase 2, APRNs continued to work in their CMS-B NHs and implemented the Payment Intervention while CMS-A implemented the Payment Intervention only. CMS-B scores in the Figures are the solid lines; B-Control scores are short dash lines; Phase 2 CMS-A scores are long dash lines; the remainder of Missouri NHs’ scores (ROTS) are medium dash lines. The restraint QM was not displayed due to the low incidence of occurrence in all groups; it was included in Phase 1 analyses (14). (Note: Lower QM scores are interpreted as “better” quality than higher scores.)

As the individual plots of the QMs illustrate, four of the eight QMs (falls, weight loss, activities of daily living, and antipsychotic medication use) for CMS-B (MOQI intervention) had more improved trajectories than most other comparison groups. For the other four QMs (urinary tract infection, pressure ulcers, restraints, and urinary catheters), the groups had mixed or similar trajectories. These results are similar to the results of the individual QM analysis in Phase 1 when CMS-B had more improvement in these same 4 QMs plus 2 additional ones, and 2 were statistically significant (ADLs and catheters) (14).

### Composite QM Score Analysis for CMS-B (MOQI APRN Intervention) and Comparison Groups

A composite QM score was calculated for each of the groups over the time of Phases 1 and 2 (2013–2019), using the same method developed and applied in the Phase 1 analysis (14). As the method was explained in Phase 1: “The composite score is the sum of the 8 long-stay QM numerators divided by the sum of the long-stay QM denominators, and then multiplied by 100. The composite QM score is a number between 0 and 100, but it is not a simple percentage because the same residents may be counted multiple times in both denominators and numerators of the 8 QMs. Because it is based on QMs, a smaller score is a better score. This method of compiling a composite score is conceptually based on the method of the calculating quality indicators (QIs), which are fore-runners of the current QMs (15°. For example, each QI was expressed as a simple ratio of the number of people in a NH with a given condition, such as weight loss, as the numerator and the number of people in the NH who could potentially have the condition, as the denominator” (14) (pg 543).

**Figure 8:**
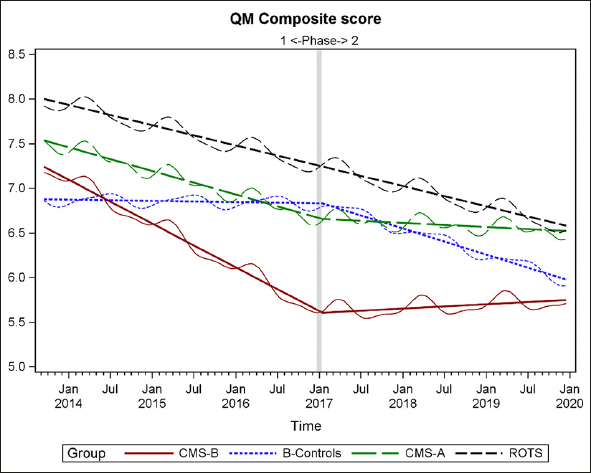
Composite of NHs and Regression Lines

Figure 8 displays the raw means (wavy lines) and the regression results (straight lines) for the four groups. Results reveal trajectories for all groups, throughout Phases 1 and 2. In Phase 2, as in Phase 1, CMS-B (MOQI Intervention + Payment) continued to maintain better quality of care as measured by QMs than B-Controls, CMS-A, and ROTS as can be seen in the Composite QM. This result is evidence of the continued effectiveness of APRNs to improve quality of care in NHs using the MOQI Intervention.

There is a Phase 2 trend of change in trajectory for groups CMS-B and CMS-A as compared to B-Controls and the remainder of the state (ROTS) in Figure 8. The downward trend (improving quality) for both CMS-B and CMS-A during Phase 1 appears to abruptly change during Phase 2 Payment Intervention and trend flat or slightly upward (not improving quality). A possible explanation for this result is that key NH staff and APRNs were so focused on implementing the Payment Intervention that direct care was adversely affected and subsequently that is reflected in poorer (flat or upward trend) quality of care QM scores.

## Discussion

In Phase 2, the analysis of QMs reveals that that the MOQI Intervention + Payment group (CMS-B) out-performed all comparison groups: B-Controls (matched to CMS-B, neither intervention), CMS-A (Payment Intervention only), and remainder of the state (ROS). These results confirm the QM analyses of Phase 1 (14), that APRNs working full-time in NHs are effective to improve quality of care. These results, with the other results of Phase 1 and 2 longitudinal analysis (20) reveal that the effectiveness of APRNs working in NHs was sustained throughout the duration of MOQI. This is important for NH leaders and policy makers to understand as they make decisions about how to encourage the use of APRNs in NHs and set new standards and regulations targeted to improve NH quality of care.

In addition to improving NH quality of care as measured by QMs (14), the MOQI Intervention achieved all primary goals of Phase 1; these were to reduce unnecessary hospitalizations and emergency department use; improve resident health outcomes; improve transitions between hospitals and NHs; and reduce healthcare spending (12). The 16 participating NHs in Phase 1 reduced potentially avoidable hospitalizations (2014–2016) by 50% and all cause hospitalizations by 32% (21). They also reduced Medicare expenditures (2014–2016) per resident per year by 40.2% for potentially avoidable hospitalizations and 28.6% for all-cause hospitalizations (12).

These important outcomes were achieved in the MOQI Intervention NHs (n=16) with full time APRNs embedded in their NHs to promote early interventions for residents with declining health conditions. The APRNs and participating NHs were supported by a Multidisciplinary Intervention Team (22) of masters/PhD prepared nurse coach, masters prepared social worker, nurse health information specialist, and medical director focused on implementing INTERACT (23), end-of-life care (23, 24, 25) health information technology (HIT) (26, 27, 28) and quality improvement using performance feedback reports (29). There are resources, including explanatory videos, provided by the research team so NHs and others who want to learn more about the successful details of the MOQI Intervention can do so https://nursinghomehelp.org/moqi-initiative/.

The improvements in transfer reductions achieved during Phase 1 were sustained longitudinally during Phase 2 for the CMS-B NHs (MOQI Intervention + Payment).20 With this Phase 2 QM analysis, it is confirmed that the intervention with APRNs also sustained quality of care improvements. However, the Payment Intervention of Phase 2 did not have the intended effect, the additional payment did not further reduce hospitalizations in CMS-B.30 Additionally, there were no significant improvements in reducing hospitalizations in CMS-A (Payment Intervention Only) (30). These results indicate that the Payment Intervention did not have the expected impact in either group.

There is an interesting finding in this QM analysis of an effect in both CMS-B and CMS-A, the two groups implementing the Payment Intervention in Phase 2 (2016–2020). There is a change in the trend of trajectory for CMS-B and CMS-A as compared to B-Controls and ROTS. Both CMS-B and CMS-A had trends of improvement in QMs evidenced by the declining slopes during the three years 2014 through 2016 (during Phase 1). Then, starting in 2017 to 2020 (during Phase 2) the trend flattens and slopes a bit upward, indicating that improvement in quality did not continue, but essentially remained the same. However, the other groups (B-Control and ROTS) moved in an improvement (decline) trajectory. There appears to be something systematic that occurred for both CMS-B and CMS-A as they implemented the Payment Intervention during Phase 2. Care in these groups may have been affected by their key NH staff focusing attention and effort on the implementation of the Payment Intervention. That shift in focus may have inadvertently negatively affected quality of care and that change was reflected in poorer quality of care QM scores.

Sustaining care in NHs appears to be a fragile effort, as other authors have discovered. While interventions can be sustained during the time of some interventions, when staff shifts focus from what have been important aspects of care, gains in progress are lost and momentum sustaining the intervention is stalled (31, 32, 33). It is important to be aware of this finding. That is one reason for NHs to employ APRNs continuously, to keep staff focused on the importance of quality care, sustaining systems such as promoting hydration, nutrition, mobility, continence, engagement in life, and early illness recognition so that early treatment is possible before much physical function is lost. Another reason is that MOQI APRNs were effective to reduce hospitalization of residents and subsequently provided significant revenue recapture for their NHs, on average $200,000 per year/200 beds (34).

There are barriers that must be removed for APRNs to work in NHs throughout the US. Changes in the Code of Federal Regulation (CFR 483.40) are needed (35). Currently, APRNs cannot bill for required visits of Medicare beneficiaries (most residents of NHs are Medicare beneficiaries) if they are hired as employees of NHs. Note that APRNs not hired by NHs may bill for these required visits. With a minor change the Federal Code, enabled by Congress, APRNs would be able to bill so that NHs could readily cover the additional salary costs of APRNs. This single change will enable nationwide hiring of APRNs by NHs, so they can improve quality of care in all NHs. Currently, NHs readily employ physicians who are also authorized to conduct and bill for required and necessary visits for skilled and long stay residents. Restricting visits by NH-employed APRNs while enabling NH-employed physicians is unnecessary regulation of an APRN’s practice. It unfairly restricts NH residents from access to APRN care. There may have been a historical concern for APRNs being pressured by NH operators to bill for unnecessary visits; CMS value-based billing now prevents this, if it is still a policy maker concern.

## Conclusions

Findings from the MOQI Initiative, both Phases 1 and 2, support that embedding APRNs in NHs, supported by an interdisciplinary team, has a positive effect on improving MDS QMs and quality of care. There are also substantial cost savings in Medicare costs. Congress enabled minor changes in federal regulations will spread these benefits to NHs nationwide. Medical Directors of NHs, all nurses and direct care staff, interdisciplinary staff, leaders, and consumers of NH care are called to come together with regulators and legislators to make these changes happen. There is no question that quality of care of NHs is in dire need of improvement. It is clearly time for action so that APRNs are working in NHs full-time to benefit the care of older adults through early illness detection, timely and appropriate treatment, and overall quality improvement. The evidence is clear and this analysis measuring the sustained effect of APRNs on QMs and quality of care is another substantial piece of that evidence.
